# A Novel Approach for Tetralogy of Fallot-Absent Pulmonary Valve Using Bioresorbable Airway Splints

**DOI:** 10.1007/s00246-024-03659-7

**Published:** 2024-09-26

**Authors:** John D. Vossler, Glenn E. Green, Andrea S. Les, Richard G. Ohye

**Affiliations:** 1 Department of Surgery, Division of Cardiovascular and Thoracic Surgery, University of California, 3020 Children’s Way, Mail Code 5078, San Diego, CA 92123 USA; 2 Department of Cardiac Surgery, Section of Pediatric Cardiovascular Surgery, University of Michigan Medical School, Ann Arbor, USA; 3 Department of Otolaryngology, Division of Pediatric Otolaryngology, University of Michigan Medical School, Ann Arbor, USA

**Keywords:** Airway, Device, Outcomes, Tracheostomy

## Abstract

Tetralogy of Fallot with absent pulmonary valve (ToF-APV) is associated with severe tracheobronchomalacia (TBM) and significant airway compromise. These patients often require early repair with right ventricle-to-pulmonary artery conduit, pulmonary arterioplasty, tracheostomy, and long-term ventilator support. A bioresorbable, 3D-printed airway splint has shown early success in treating severe TBM and has the potential to obviate the need for early repair with conduit and tracheostomy. A retrospective case series analysis was conducted on consecutive patients with ToF-APV and severe TBM who underwent airway splinting between 2012 and 2021. Clinical data was extracted from the medical record. Patients were grouped and analyzed according to their sequence of procedures. Eight patients with ToF-APV and severe TBM underwent airway splinting with a median follow up of 3.6 years (range 1.0–6.4). All patients were alive at the most recent follow-up. Five patients underwent complete cardiac repair first, and one patient underwent concurrent complete cardiac repair and airway splinting. All six of these patients required tracheostomy and long-term ventilator support, even after airway splinting. Five of six remained on ventilator support at the most recent follow up. Two patients underwent airway splinting before complete cardiac repair. Neither required tracheostomy nor prolonged ventilatory support (*p* = 0.036). Both were discharged home in the neonatal period and returned as infants to undergo elective ToF-APV repair. Patients with TOF-APV and severe TBM may be able to avoid early repair with conduit placement and tracheostomy by undergoing airway splinting prior to complete cardiac repair.

## Introduction

Tetralogy of Fallot with absent pulmonary valve (ToF-APV) is a rare form of ToF that is often associated with DiGeorge syndrome [1–4]. These patients have rudimentary or absent pulmonary valve leaflets and stenosis of the pulmonary annulus leading to pulmonary stenosis and pulmonary insufficiency, which causes aneurysmal dilation of the pulmonary arteries (PAs) [2–5]. ToF-APV patients may present in the neonatal period with severe, multifactorial respiratory insufficiency from mechanical compression of the airways by the aneurysmal pulmonary arteries and associated tracheobronchomalacia (TBM) [1–4].

Tracheobronchomalacia is characterized by airway cartilage that lacks structural integrity leading to dynamic airway collapse with normal respiration [6]. Limited treatment options exist for severe pediatric TBM. Standard therapies include tracheostomy with long-term ventilator support, aortopexy, tracheobronchopexy, and intraluminal metallic, silicone, or bioresorbable stents [4, 6]. Despite these treatment options, many patients require tracheostomy with long-term ventilatory support, and the mortality rate for severe pediatric TBM exceeds 50% [4, 6].

A 3D-printed, bioresorbable airway splint was developed to address this problem [6–8]. While pursuing an Investigational Device Exemption from the Food and Drug Administration (FDA) that would permit a clinical trial, children with severe TBM were treated with these splints at the University of Michigan under FDA Expanded Access (Emergency Use and Compassionate Use), with appropriate approvals from the FDA and Institutional Review Board. During this time period, several patients with ToF-APV and severe TBM were treated with airway splinting. It was observed that patients who underwent airway splinting prior to complete repair of ToF-APV were able to avoid tracheostomy and long-term ventilator support. The purpose of this study is to compare the clinical course of patients with ToF-APV and severe TBM who underwent airway splinting prior to complete repair to those who underwent airway splinting after complete repair.

## Patients and Methods

### Design, Data Source, and Patients

A retrospective case series analysis was conducted. Patients were identified by querying a prospectively maintained registry of patients who have received airway splints. Patients with diagnoses of ToF-APV and severe TBM who underwent airway splinting between January 1, 2012 and December 31, 2021 were included. No exclusion criteria were applied. Data was gathered from the University of Michigan electronic medical record by manual chart review. This study was reviewed and approved by the University of Michigan Institutional Review Board and deemed exempt from requiring individual patient consent (HUM00223004).

### Airway Splinting

The fabrication and implantation of airway splints has been described previously [6–8]. Briefly, bioresorbable airway splints are 3D-printed by laser sintering from a mixture of 96% polycaprolactone and 4% hydroxyapatite. The splints are a 270 tubular structure with rows and columns of fenestrations. The structure is rigid to provide resistance against collapse while also being flexible to allow for movement and growth. The material is estimated to resorb in 2–4 years. Splint implantation is done via median sternotomy. Cervical incision and cardiopulmonary bypass are used as needed to facilitate exposure of the airway. Intraoperative bronchoscopy is performed to delineate the diseased segment of airway. A series of partial thickness polypropylene sutures are placed around the anterior 270° of the airway along the length of the diseased segment. The sutures are passed through the splint fenestrations, the splint is “parachuted” onto the airway, and the sutures are secured. This suspends the airway within the splint. Post-implant bronchoscopy is performed to confirm resolution of disease. Splints can be implanted on the trachea, right mainstem bronchus, and/or left mainstem bronchus.

### Analysis

Patient characteristic variables analyzed were demographics, diagnoses, comorbidities, pre-operative risk factors, and clinical course. Patients were split into cohorts based on the timing of airway splinting relative to ToF-APV repair. Cohort 1 patients underwent airway splinting after or during ToF-APV repair. Cohort 2 patients underwent airway splinting before ToF-APV repair. Short-term outcomes analyzed were 30-day morbidity and mortality following ToF-APV repair. Long-term outcomes analyzed were mortality and requirement for tracheostomy and long-term ventilator support following the most recent surgical procedure. Fisher’s exact test was used for inferential statistics. Statistical significance was assigned to *p*-value < 0.05. Statistical analysis was performed using R version 4.3.0.

## Results

### Demographics and Comorbidities

Demographics and comorbidities of cohorts 1 and 2 are reported in Table 1.A total of 8 patients were included in the study. Cohort 1 (splint after/during ToF-APV repair) had 6 patients, and cohort 2 (splint before ToF-APV repair) had 2 patients. There was no difference in sex, prematurity, DiGeorge syndrome, chromosomal abnormality, pre-operative extracorporeal membrane oxygenation requirement, or gastrostomy tube dependence between cohorts.

**Table 1:** Demographics and comorbidities of cohorts 1 and 2

Characteristic	All patients n (%)	Cohort 1 Splint after/during ToF-APV repair n (%)	Cohort 2 Splint before ToF-APV repair n (%)	p-value^i^
Number of patients	8 (100%)	6 (75%)	2 (25%)	–
Female	3 (38%)	2 (33%)	1 (50%)	1.000
Premature (GA < 36 weeks)	1 (13%)	0 (0%)	1 (50%)	0.250
DiGeorge syndrome	5 (63%)	4 (67%)	1 (50%)	1.000
Any chromosomal abnormality	7 (88%)	5 (83%)	2 (100%)	1.000
ECMO required before first operation	1 (13%)	1 (17%)	0 (0%)	1.000
G-tube dependence	5 (63%)	5 (83%)	0 (0%)	0.107

i, Fisher’s exact test. ToF-APV, Tetralogy of Fallot with absent pulmonary valve; *GA*, gestational age; *ECMO*, extracorporeal membrane oxygenation; *G*-tube, gastrostomy tube.

### Clinical Course and Operative Characteristics

Clinical course and operative characteristics for cohort 1 (airway splint after/during ToF-APV repair) are reported in Table 2. Patients 1–5 underwent ToF-APV repair at a median age of 20 days (range, 7–46 days). Four patients had a right ventricle (RV) to PA conduit for their right ventricular outflow tract (RVOT) reconstruction, and one patient had a transannular patch. Three patients had bilateral branch PA plication, one had only right PA plication, and one did not have PA plication. Three patients had a LeCompte maneuver as part of their repair. All five patients required interval tracheostomy placement at a median age of 72 days (range, 42–120 days) followed by airway splinting at a median age of 173 days (range, 154–823 days). Three patients required CPB (cardiopulmonary bypass) for splint placement. Three patients had airway splints placed on the trachea and bilateral mainstem bronchi. One patient had splints placed on the bilateral mainstem bronchi only, and one had splints placed on the left mainstem bronchi only. Patient 6 underwent airway splinting during ToF-APV repair at age 337 days. She underwent tracheostomy placement at age 13 days, had an RV-PA conduit RVOT reconstruction without PA application or LeCompte maneuver, and received only a right mainstem bronchi splint.

**Table 2:** Clinical course and operative characteristics for cohort 1 (airway splint after /during ToF-APV repair)

Patient	ToF-APV Repair				Interval (days)	Tracheostomy	Interval (days)	Airway Splint				
	Age (days)	RVOT Reconstruction	Branch PA Plication	LeCompte Maneuver		Age (days)		Age (days)	CPB	Trachea	Right Mainstem	Left Mainstem
Patient 1	20	RV-PA conduit	Bilateral	No	52	72	442	514	Yes	No	Yes	Yes
Patient 2	7	TAP w/ GoreTex monocusp	Right	No	113	120	53	173	No	No	No	Yes
Patient 3	28	RV-PA conduit	None	Yes	58	86	82	168	Yes	Yes	Yes	Yes
Patient 4	46	RV-PA conduit	Bilateral	Yes	24	70	753	823	Yes	Yes	Yes	Yes
Patient 5	14	RV-PA conduit	Bilateral	Yes	28	42	112	154	No	Yes	Yes	Yes
Patient 6	337	RV-PA conduit	None	No	-324	13	324	337	No	No	Yes	No

*ToF-APV*, Tetralogy of Fallot with absent pulmonary valve; *RVOT*, right ventricular outflow tract; *RV*, right ventricle; *PA*, pulmonary artery; *TAP*, transannular patch; *CPB*, cardiopulmonary bypass

Clinical course and operative characteristics for cohort 2 (airway splint before ToF-APV repair) are reported in Table 3. Patient 7 underwent initial palliation with an RV-PA conduit, internal PA band, and bilateral branch PA application at age 49 days followed 3 days later by splint of the left mainstem bronchus with CPB. Patient 8 did not require initial palliation, and his first procedure was splinting of the bilateral mainstem bronchi with CPB. Both patients were discharged home. Patient 7 returned at age 432 days for complete ToF-APV repair with replacement of the previously placed RV-PA conduit and no further PA intervention, and patient 8 returned at age 289 days for complete ToF-APV repair with transannular patch and no pulmonary artery intervention. Neither patient underwent tracheostomy placement.

**Table 3:** Clinical course and operative characteristics for cohort 1 (airway splint before ToF-APV repair)

After Patients	Initial Palliation		Interval (days)	Airway Splint					Interval (days)	ToF-APV Repair			
	Age (days)	Operation		Age (days)	CPB	Trachea	Right Mainstem	Left Mainstem		Age (days)	RVOT Reconstruction	Branch PA Plication	LeCompte Maneuver
Patient 7	49	RV-PA conduit Internal PA band Bilateral branch PA plication	3	52	Yes	No	No	Yes	380	432	RV-PA conduit replacement	Bilateral (prior)	No
Patient 8	None		-	49	Yes	No	Yes	Yes	240	289	TAP	None	No

*ToF-APV*, Tetralogy of Fallot with absent pulmonary valve; *RVOT*, right ventricular outflow tract; *RV*, right ventricle; *PA*, pulmonary artery; *TAP*, transannular patch; *CPB*, cardiopulmonary bypass

### Outcomes

Short- and long-term outcomes for both cohorts are reported in Table 4. There was no difference in 30-day mortality, cardiac arrest, dysrhythmia, surgical site infection, central line-associated bloodstream infection, reintubation, diaphragm paresis, or chylothorax. At a median follow up of 3.6 years (interquartile range 1.0–6.4 years) there was no mortality in either group. All six patients in cohort 1 (airway splint after/during ToF-APV repair) required tracheostomy placement and long-term ventilator support whereas no patients in cohort 2 (airway splint before ToF-APV repair) required this (*p* = 0.036). One patient in cohort 1 has since been decannulated.

**Table 4:** Short- and long-term outcomes of cohorts 1 and 2

Characteristic	All patients n (%)	Cohort 1 Splint after/during ToF-APV repair n (%)	Cohort 2 Splint before ToF-APV repair n (%)	p-value^i^
Number of patients	8 (100%)	6 (75%)	2 (25%)	–
30-Day Outcomes Following ToF-APV Repair				
Mortality	0 (0%)	0 (0%)	0 (0%)	1.000
Cardiac arrest	1 (13%)	1 (17%)	0 (0%)	1.000
Dysrhythmia	2 (25%)	2 (33%)	0 (0%)	1.000
Surgical site infection	2 (25%)	2 (33%)	0 (0%)	1.000
CLABSI	1 (13%)	1 (17%)	0 (0%)	1.000
Reintubation	2 (25%)	1 (17%)	1 (50%)	0.464
Diaphragm paresis	1 (13%)	0 (0%)	1 (50%)	0.250
Chylothorax	1 (13%)	0 (0%)	1 (50%)	0.250
Long-Term Outcomes Following Most Recent Procedure				
Follow up (years), median (range)	3.56 (0.96–6.41)	4.20 (1.07–6.41)	2.09 (0.96–3.22)	–
Mortality	0 (0%)	0 (0%)	0 (0%)	1.000
Tracheostomy & long-term ventilator	6 (75%)	6 (100%)	0 (0%)	0.036

i, Fisher’s exact test. *ToF-APV*, Tetralogy of Fallot with absent pulmonary valve; *CLABSI*, central line-associated blood stream infection

### Comment

This study reports the clinical course of patients with ToF-APV and severe TBM who underwent airway splinting. Patients who underwent airway splinting prior to complete ToF-APV repair were not only able to avoid tracheostomy and long-term ventilator support entirely but they were also able to be discharged home in the neonatal period and returned later in infancy for scheduled, elective, complete repair of ToF-APV. One of these patients was also able to be repaired without the use of an RV-PA conduit, thus minimizing the patient’s risk of the need for re-intervention.

Current surgical approaches to patients with ToF-APV with respiratory failure focus on relieving the mechanical compression of the airways by the aneurysmal PAs by techniques that include anterior/posterior plication of aneurysmal PAs, excision of PA wall segments, suspension of the PA to the retro sternal fascia, translocating the PA anterior to the aorta with Lecompte maneuver, or complete replacement of the mediastinal PA with a bifurcated pulmonary homograft [3]. These approaches have had limited success because they fail to address the TBM associated with ToF-APV. The use of airway splints in these patients serves the dual purpose of protecting the airways from mechanical compression from the PAs as well as supporting the airways from dynamic airway collapse from TBM.

Natural airway growth and maturation will generally resolve the symptoms of TBM after approximately 24 months of support [6]. This support typically takes the form of positive pressure ventilation delivered by tracheostomy and long-term ventilator support, which is well-known to be associated with significant morbidity. Prior attempts at external airway splinting showed promising early results at avoiding tracheostomy, but were limited by the lack of bioresorbable materials [9]. The splints used in this study are estimated to resorb in 2–4 years [7], which is hypothesized to be long enough to support the airways until natural resolution of TBM.

The novel approach to ToF-APV reported in this paper refers to the order of operations. We hypothesize that treating the associated TBM first with early airway splinting will not only allow these patients to avoid tracheostomy and long-term ventilator support but also allow them to return to the typical treatment pathway and risk profile of other ToF patients. This approach may allow ToF-APV patients to have a transannular patch alone for their RVOT reconstruction and avoid complex PA angioplasty, LeCompte maneuver, and RV-PA conduit placement. Although Patient 7 had undergone a procedure to alleviate PA compression, including an RV-PA conduit, it is possible that he would not have required this initial intervention had splinting been the index operation. Regardless, he did avoid the need for tracheostomy and long-term ventilation experienced by all of the patients in cohort 1.

This study has several limitations. First, its retrospective design precludes conclusions of causality. Next, these results represent the early experience of a single-center using a novel device and may not be generalizable. Next, this study is of a small number of patients not enrolled in a clinical trial, and thus lacks the power to accurately detect even large differences in outcomes. Finally, as is the case with all retrospective analyses, there are several relevant data points that are not available, not detailed, or incomplete. The patients included in this study were mostly referred from other institutions and received most of their pre- and post-operative care elsewhere, thus there are many missing relevant details.

Patients with ToF-APV and severe TBM may be able to avoid tracheostomy, long-term ventilator support, and complex RVOT reconstruction, including RV-PA conduit placement, by undergoing early airway splinting as their initial treatment. Further study is necessary to evaluate the safety and efficacy of these airway splints as well as this novel approach to ToF-APV.

## Data Availability

No datasets were generated or analysed during the current study.

## Abbreviations

ToF-APV: Tetralogy of Fallot with absent pulmonary valve
TBM: Tracheobronchomalacia
FDA: Food and Drug Administration
PA: Pulmonary artery
RV: Right ventricle
RVOT: Right ventricular outflow tract
CPB: Cardiopulmonary bypass
